# Comprehensive Characterization of Arterial and Cardiac Function in Marfan Syndrome—Can Biomarkers Help Improve Outcome?

**DOI:** 10.3389/fphys.2022.873373

**Published:** 2022-04-25

**Authors:** Constance G. Weismann, Joanna Hlebowicz, Anna Åkesson, Petru Liuba, Katarina Hanseus

**Affiliations:** ^1^ Department of Clinical Sciences, Lund University, Lund, Sweden; ^2^ Department of Pediatric Cardiology, Pediatric Heart Center, Skåne University Hospital, Lund, Sweden; ^3^ Department of Pediatric Cardiology and Pediatric Intensive Care Medicine, Ludwig-Maximilian University Hospital, Munich, Germany; ^4^ Department of Cardiology, Skåne University Hospital, Lund University, Lund, Sweden; ^5^ Clinical Studies Sweden - Froum South, Skåne University Hospital, Lund, Sweden

**Keywords:** Marfan syndrome, augmentation index, pulse wave velocioty, arterial stiffness, cardiac function, endothelial function, intima media thickness, brain natriuretic peptide (BNP)

## Abstract

**Background:** Marfan Syndrome (MFS) has been associated with increased aortic stiffness and left ventricular dysfunction. The latter may be due to the underlying genotype and/or secondary to aortic stiffening (vascular-ventricular interaction). The aim of this study was to characterize arterial and cardiac function in MFS using a multimodal approach.

**Methods:** Prospective observational study of MFS patients and healthy controls. Methods included echocardiography, ascending aortic distensibility, common carotid intima media thickness [cIMT], parameters of wave reflection, carotid-femoral pulse wave velocity [cfPWV]), reactive hyperemia index [RHI], and biomarker analysis (Olink, CVII panel).

**Results:** We included 20 patients with MFS and 67 controls. Ascending aortic distensibility, cIMT and RHI were decreased, while all parameters of arterial wave reflection, stiffness and BNP levels were increased in the MFS group. Both systolic and diastolic function were impaired relative to controls. Within the MFS group, no significant correlation between arterial and cardiac function was identified. However, cfPWV correlated significantly with indexed left ventricular mass and volume in MFS. Bran natriuretic peptide (BNP) was the only biomarker significantly elevated in MFS following correction for age and sex.

**Conclusions:** MFS patients have generally increased aortic stiffness, endothelial dysfunction and BNP levels while cIMT is decreased, supporting that the mechanism of general stiffening is different from acquired vascular disease. CfPWV is associated with cardiac size, blood pressure and BNP in MFS patients. These may be early markers of disease progression that are suitable for monitoring pharmacological treatment effects in MFS patients.

## Introduction

Marfan syndrome (MFS) is an autosomal-dominant connective tissue disorder, most commonly caused by missense mutations in the *FBN1* gene. It is a rare disease with a prevalence of 6.5/100,000 in Sweden. (Groth et al., 2015). Long-term survival is largely defined by cardiovascular complications. Patients with haplo-insufficient *FBN1* mutations have a 2.5-fold increased risk for cardiovascular mortality compared to carriers of dominant-negative *FBN1* mutations. (Franken et al., 2016). MFS is diagnosed based on the revised Ghent criteria from 2010, which includes clinical and genetic criteria as well as the family history. (Loeys et al., 2010). The cardiovascular phenotype of MFS is variable. The most well-described and prognostically highly relevant cardiovascular features are 1) aortic root dilation with the risk of aortic regurgitation and - most importantly—aortic dissection as well as 2) mitral valve prolapse with resulting mitral insufficiency and possible chordal rupture. As FBN1 is an ubiquitously expressed elastin-associated glycoprotein of the extracellular matrix, it is not surprising that the phenotypic expression of *FBN1* mutations is wide-spread. Increased aortic stiffness has been described *in vitro*, in animal models as well as in humans. (De Backer et al., 2006; Syyong et al., 2009; Kiotsekoglou et al., 2011; Crosas-Molist et al., 2015; Salvi et al., 2018). Further, endothelial dysfunction has also been described by some, though agonist mediated endothelium-dependent vasodilation appears intact. (Wilson et al., 1999; Nakamura et al., 2000; Chung et al., 2007; Syyong et al., 2009).

In addition to vascular abnormalities, many patients with MFS have impaired systolic as well as diastolic cardiac function. It remains unknown whether cardiac dysfunction is a result of adverse central hemodynamics due to vascular-ventricular interaction, or whether it is a primary consequence of the underlying genetic defect. A mouse model has shown *in vivo* that PWV is increased and correlates with age in Fbn1C1039G/+ mice, but not wild-type mice. (Lee et al., 2016). Further, systolic and diastolic function were decreased and *in vitro* analysis revealed increased left ventricular (LV) size and mass. To our knowledge, a similar comprehensive longitudinal assessment of cardiac, and vascular characterization has not been performed in humans.

Clinical trials using beta blockers and angiotensin II receptor blockers (ARB) or ACE inhibitors have focused on progression of aortic root dilation. A recent meta-analysis of seven studies that included a total of 1,510 patients confirmed that use of angiotensin II receptor blockers is associated with slower progression of aortic root dilation when using it as a mono-therapy or in addition to beta-blocker therapy. (Al-Abcha et al., 2020). By contrast, only a few small pharmacological clinical trials have focused on changes in arterial stiffness, which in the general population with acquired cardiovascular disease is an important marker of outcome. (Ahimastos et al., 2007; Bhatt et al., 2015).

We herein describe a multi-modal approach to analyze vascular, cardiac, and biochemical characteristics in children and adults with MFS compared to a matched control group. It was the primary aim of this cross-sectional study to provide evidence whether cardiac dysfunction in MFS may result from arterial stiffness by means of vascular-ventricular interaction. The secondary aim was to evaluate cardiovascular function in the context of age. Lastly, our aim was to identify biomarkers associated with MFS.

We hypothesized that Marfan syndrome patients have a general arteriopathy and endothelial dysfunction which correlate with the degree of systolic and diastolic cardiac dysfunction. Further, we hypothesized that arterial and cardiac abnormalities worsen with increasing age and that MFS patients have a biomarker expression pattern distinct from controls.

## Methods

This is a prospective observational cohort study comparing cardiovascular function in patients with Marfan syndrome (MFS group) compared to healthy controls (Control group) using a multimodal approach. The study was conducted 2017–2019. The study was approved by the local ethics committee (#2017/243).

MFS patients were recruited through the Swedish Registry for Congenital Heart Diseases (SWEDCON). Healthy controls were recruited through advertisement. All MFS patients had a confirmed diagnosis based on revised Ghent criteria and included patients with prior aortic surgery. (Loeys et al., 2010). MFS group specific exclusion criteria were more than moderate aortic regurgitation. Exclusion criteria for the control group were cardiovascular disease or hypertension. General exclusion criteria were systemic inflammatory disorders, diabetes and cancer.

Baseline characteristics such as gender, age, weight, height, body surface area, body mass index, blood pressure and heart rate were recorded. For the MFS group, genetic testing, phenotypic complications, and prior cardiac and/or aortic surgery if applicable were documented as well.

The following methods were employed; protocols used were described in detail previously: (Weismann et al., 2021):

1 Echocardiograms including 2-dimensional, color, spectral, tissue Doppler (TDI) and 4-dimensional imaging. Z-scores for aortic dimensions were determined. (Lopez et al., 2017).
2 Ultrasound of the ascending aorta to determine distensibility and strain
3 Common carotid artery ultrasound to determine average right and left intima media thickness (cIMT) and right sided common carotid lumen dimension, distensibility, stiffness index and strain.
4 Carotid-femoral arterial pulse wave velocity (PWV), central blood pressure and augmentation index (cAIx) corrected to a heart rate of 75 beats per minute (cAIx75), were determined using SphycmoCor XCEL (AtCor, Australia). (Butlin and Qasem, 2017).
5 Peripheral augmentation index corrected to a heart rate of 75 beats per minute (pAIx75) was obtained using EndoPAT 2000 (Itamar Medical, Israel)
6 Aging index (AI) is derived from the acceleration curve obtained by photoplethysmography based digital pulse wave analysis (DPA; Meridian, South Korea). A higher, less negative AI is consistent with aging, i.e., stiffer arteries.
7 Reactive hyperemia index (RHI), a marker of endothelial function, was assessed using EndoPAT 2000 (Itamar Medical, Israel). (Hamburg et al., 2008; Rubinshtein et al., 2010). The peripheral augmentation index ((P2-P1)/P1) corrected to a heart rate of 75 beats per minute (pAIx75) was derived.

In addition, blood samples were obtained by venipuncture, placed in an EDTA tube, frozen immediately and stored at −80°C. One μl of each blood sample was analyzed with the 92-biomarker “Cardiovascular II” panel by the Proximity Extension Assay (PEA) technique. Analyses were performed at the Clinical Biomarkers Facility, Science for Life Laboratory, Uppsala, using the Proseek Multiplex CVD96 × 96 reagents kit (Olink Bioscience, Uppsala, Sweden).

### Statistics

For statistical analyses, continuous variables were expressed as median and inter-quartile range (IQR). Categorical variables were expressed as frequency. Group comparisons were made using Mann-Whitney U and Fisher exact tests as appropriate. Univariate regression analyses were carried out correcting for possible covariates, including age, sex, height, and heart rate. Only covariates associated with the outcome variable (defined as a *p* < 0.1) were then included in the model as outlined in the respective tables legends. A *p*-value of <0.05 was considered statistically significant. Variables were also associated using Pearson’s correlation coefficient (r). For biomarkers, *p*-values were adjusted using the Benjamini & Hochberg correction (false discovery rate). Data were stored using REDCap electronic data capture tools hosted at Lund University. Statistical analysis was performed using Statistical Package for Social Sciences, version 27 (IBM SPSS, Chicago, IL).

## Results

We prospectively recruited 20 patients with a history of MFS and 71 healthy controls between the ages 9 and 49 (median age 25). Four controls were excluded due to incomplete data acquisition for technical reasons.

### MFS Cohort Description

Nineteen (95%) patients had a history of aortic root dilation, 1 (5%) aortic dissection, 9 (45%) mitral valve prolapse, 3 (15%) lens dislocation, 1 (5%) scoliosis requiring surgery, 2 (10%) dural ectasia, and 0 (0%) had had a spontaneous pneumothorax. Genetic testing had been performed in 17 patients, of which 16 (80%) had a presumed pathogenic mutation in *FBN1*. Five (25%) patients had previously undergone aortic root replacement (3 valve sparing, 2 mechanical aortic valves). No aortic re-interventions have been performed. One patient (5%) had a mechanical mitral valve.

At the time of the study visit, no one had aortic stenosis or more than mild aortic insufficiency (5 trivial, 2 mild). The two patients with mild aortic insufficiency had pressure half-times of 461 and 864 msec, respectively. Abdominal aortic PW Doppler (technically feasible in 18 of 20 patients) was interpreted as normal in all. The majority of MFS was treated with either the angiotensin II receptor blocker Losartan, the beta_1_ receptor blocker Metoprolol (total *n* = 16, 80%), or both (*n* = 5, 25%).

### Basic Data

Other than MFS patients being taller than controls, as expected, there was no significant difference in other baseline demographics such as age, sex, weight, body surface area (BSA), heart rate, and brachial blood pressure (Table 1). There was a trend towards a higher body mass index (BMI) in the control group (*p* = 0.066, Table 1).

**TABLE 1:** Descriptive statistics comparing basic characteristics and outcome variables of Controls to MFS patients Continuous variables are presented as median (interquartile range) and categorical variables as percent (number).

	Controls (*n* = 67)	MFS (*n* = 20)	*p*-value*
**Basic Characteristics**			
Age [years]	25 (20–35)	22 (12–37)	0.452
Male sex	54% (36)	50% (10)	0.803
Height [cm]	173 (163–182)	179 (174–191)	0.038*
Weight [kg]	70 (58–80)	62 (49–89)	0.916
BMI [kg/m^2^]	23.1 (21.0–24.8)	20.5 (16.3–23.9)	0.066
BSA [m^2^]	1.83 (1.66–2.03)	1.80 (1.52–2.18)	0.635
Systolic BP [mmHg]	118 (111–123)	119 (105–123)	0.577
Diastolic BP [mmHg]	68 (64–75)	73 (66–75)	0.536
Heart rate [1/min]	60 (53–68)	59 (56–65)	0.553
**Cardiac function and structure**			
E’ [cm/s]	13.6 (12.1–15.1)	11.0 (9.4–12.0)	<0.001*
E/E’ [cm/s]	5.7 (5.0–6.4)	6.9 (5.4–8.3)	0.021*
Ejection Fraction [%]	62.3 (59.7–64.5)	51.8 (50.1–56.6)	<0.001*
Global longitudinal strain [%]	−20.8 (−22.3 to −19.7)	−16.4 (−18.1 to −13.7)	<0.001*
LV mass/BSA [g/m^2^]	67.8 (50.8–78.9)	72.4 (60–115)	0.038*
LV EDV/BSA [ml/m^2^]	59.8 (54–68)	71.9 (58.6–92.1)	0.009*
**Vascular characteristics**			
Aortic dimensions (mm/Z-score)			
Valve annulus^#^	2.1 (1.8–2.4)/0.6(−0.3–1.4)	2.2(2–2.5)/1.5(1.1–2.3)	0.169/0.014*
Root^#^	2.9 (2.6–3.2)/0.5 (-0.3–1.1)	3.8 (3.2–4.2)/4.3(3.1–4.8)	<0.001*/<0.001*
Sinotubular junction^#^	2.4 (2.1–2.6)/0.4(−0.4–1.4)	2.7 (2.4–3.2)/2.1(1.4–3)	0.017*/<0.001*
Ascending aorta	2.8(2.5–3.1)/1.4(0.7–2.1)	2.6(2.3–2.9)/1.1(0.5–1.6)	0.182/0.208
Ascending aortic elasticity			
Distensibility	5.6 (4.4–7.2)	2.5 (1.5–4.6)	<0.001*
Stiffness Index	4.0 (3.0–5.1)	8.7 (5.3–13.2)	<0.001*
Strain [%]	13.0 (10.5–17.9)	5.8 (3.8–8.4)	<0.001*
Common carotid artery			
Lumen dimension [mm]	6.4 (6.1–6.9)	6.6 (6.2–7.1)	0.305
Distensibility	5.3 (4.4–6.6)	5.4 (4.4–6)	0.862
Stiffness Index	4.2 (3.4–5)	4.1 (3.7–4.9)	0.983
Strain [%]	13.3 (10.3–15.9)	11.9 (10.3–13.6)	0.342
cIMT [mm]	0.47 (0.43–0.51)	0.43 (0.41–0.46)	0.039*
Functional arterial characteristics			
Central Systolic Pressure [mmHg]	102 (95–108)	103 (91–107)	0.916
Central Diastolic Pressure [mmHg]	69 (65–77)	73 (64–75)	0.758
cAix75%	−7.6 (−15.5–0.1)	−0.7 (−7.8–7.7)	0.011*
Augmentation pressure [mmHg]	0.5 (-2.5–2.5)	1.8 (-0.3-6)	0.029*
pAIx75%	−20 (−27 to −10)	−9 (−17–7)	0.002*
Aging Index	−0.8 (−1 to −0.6)	−0.7 (−0.8 to −0.2)	0.008*
Pulse Wave Velocity [m/s]	6.4 (5.0–7.1)	7.5 (6.0–8.7)	0.004*
Reactive Hyperemia Index	2.1 (1.6–2.6)	2.1 (1.4–2.3)	0.098

Groups were compared using the Mann-Whitney test or Fisher’s exact test as appropriate. Significance level (p) is provided. * marks *p* < 0.05.^#^ excluding 5 MFS patients who have previously undergone aortic root replacement.

### Cardiac Findings

Focusing on cardiac function, diastolic function was significantly impaired compared to healthy controls (Table 1). This was evidenced by a lower average E′ velocity by TDI and a higher average E/E’ ratio. Systolic function described by 4-dimensional EF was roughly 10% points lower in MFS patients compared to controls (Table 1). Likewise, global longitudinal strain was less negative in the MFS cohort. Structurally, LV mass, and volume normalized to body surface area were both significantly increased in the MFS group compared to controls. There was a strong correlation between the two, and in a linear regression analysis LV mass index was not different between the groups when correcting for LV volume index (*p* = 0.292). For the subgroup of patients who have not had aortic root replacement, aortic root and sinotubular junction Z-scores were significantly increased in the MFS group, while for the aortic valve Z-score but not absolute dimension met criteria for statistical significance. Ascending aortic dimension and Z-score were similar between patients and controls.

### Arterial Function

Central blood pressure was comparable to the control group (Table 1). However, the MFS group consistently revealed pathologic changes of arterial stiffness and wave reflection, even following correction for covariates (Tables 1, 2). This was evidenced by decreased ascending aortic elasticity parameters as well as increased cAIx75, pAIx75, AI, and cfPWV. By contrast, cIMT was decreased compared to controls and there was no evidence of increased common carotid artery stiffness (Tables 1, 2). Lastly, RHI, which is a marker of endothelial function, revealed no significant difference between the MFS and control groups using the non-parametric test (Table 1). However, when correcting for confounders, RHI was significantly lower in the MFS group (Table 2), which is consistent with impaired endothelial function. When adding use of angiotensin II receptor blockers–which can augment endothelial function—to the model as a co-variate, the results were maintained (B = −0.54 (95% CI −1 to −0.08), *p* = 0.022). Group differences of vascular characteristics were maintained when excluding MFS patients with prior aortic root replacement (data not shown).

**TABLE 2:** Linear regression model comparing vascular outcome variables of MFS patients to Controls.

	MFS Vs. control B (95% CI)	p
AscAo Distensibility^$^	−3.0 (-4.1 to −1.9)	<0.001*
CCA IMT [mm]^$^	−0.030 (−0.057 to −0.003)	0.029*
cAix75%^$§^	14.1 (7.6–20.7)	<0.001*
pAIx75%^$§^	20.1 (13.1–27.2)	<0.001*
Aging Index^$§*^	0.35 (0.24–0.46)	<0.001*
Pulse Wave Velocity [m/s]^$§^	1.4 (0.7–2.1)	<0.001*
Reactive Hyperemia Index^!§^	−0.45 (-0.79 to −0.10)	0.011*

B (95% confidence interval) corrected for significant covariates are shown. ^$^ age ^!^ sex * heart rate, § height.

### Vascular-Ventricular Interaction

**FIGURE 1:**
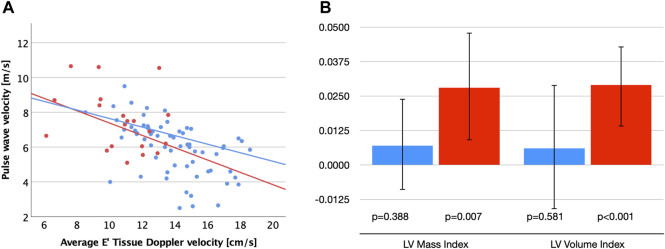
**(A)** Scatter plot visualizing correlations between carotid-femoral pulse wave velocity (cfPWV) and diastolic function (E′) for patients with MFS (red dots and regression line) and healthy controls (blue dots and regression line). **(B)** Unstandardized B-coefficients for associations between cfPWV with left ventricular (LV) mass and volume index corrected for age and sex. Error bars indicate 95% confidence intervals. MFS (red), Control group (blue).

Next, we evaluated whether cardiac function correlates with arterial function (Table 3; Figure 1). Overall, diastolic function—represented herein by TDI derived average E’—showed moderate-strong and highly significant negative correlations with the following arterial characteristics in a descending order (absolute values for r): cfPWV (Figure 1A), inverse ascending aortic distensibility, parameters of wave reflection (cAIx, pAIx, AI), and CSP. However, within the MFS group, none of the parameters correlated with E’ even when correcting for cardiovascular medications and prior aortic surgery (data not shown). All results overall and in the individual groups were maintained when performing partial correlations controlling for age and sex (data not shown).

**TABLE 3:** Correlations between cardiovascular characteristics and average E’ (diastolic function), EF (systolic function), or age for all patients as well as group-wise analysis for controls and MFS patients.

	All		Control		MFS	
	R	P	R	p	R	p
**Average E’**						
AscAo Distensibility	0.455	<0.001*	0.314	<0.011*		0.339
CCA IMT [mm]	−0.256	0.017*	−0.501	<0.001*		0.279
Central systolic pressure [mmHg]	−0.310	0.004*	−0.564	<0.001*	−0.429	0.059
cAIx75%	−0.406	<0.001*	−0.382	0.002*		0.802
pAIx75%	−0.343	0.002*	−0.230	0.069		0.429
Aging Index	−0.365	<0.001*		0.288	−0.384	0.094
Pulse Wave Velocity [m/s]	−0.567	<0.001*	−0.523	<0.001*	−0.307	0.188
**Average EF**						
AscAo Distensibility	0.422	<0.001*	0.234	0.059		0.287
CCA IMT [mm]		0.720		0.230		0.285
Central systolic pressure [mmHg]		0.673	−0.354	0.003*		0.837
cAIx75%	−0.275	0.011*		0.278		0.441
pAIx75%	−0.243	0.027*		0.214		0.804
Aging Index	−0.261	0.016*		0.401		0.530
Pulse Wave Velocity [m/s]	−0.326	0.002*		0.406		0.419
**Age**						
AscAo Distensibility	−0.353	0.001*	−0.437	<0.001*	−0.561	0.015*
CCA IMT [mm]	0.559	<0.001*	0.574	<0.001*	0.606	0.005*
Central systolic pressure [mmHg]	0.354	<0.001*	0.360	0.003*		0.372
cAIx75	0.396	<0.001*	0.506	<0.001*	0.532	0.019*
pAIx75	0.301	0.006*	0.345	0.005*	0.382	0.106
Aging Index	0.316	0.003*	0.297	0.017*	0.556	0.011*
Pulse Wave Velocity [m/s]	0.521	<0.001*	0.597	<0.001*	0.600	0.005*
E’ [cm/s]	−0.428	<0.001*	−0.573	<0.001*	−0.502	0.024*
Ejection fraction [%]		0.279		0.215	−0.341	0.142

Pearson’s correlation coefficient R is reported if *p* < 0.2. * marks all *p* < 0.05.

Repeating these correlation analyses with EF, describing systolic function, we identified weak-moderate negative correlations with the following arterial characteristics in a descending order (absolute values for r): inverse ascending aortic distensibility, cfPWV, parameters of wave reflection (cAIx, pAIx, and AI). CSP did not correlate significantly with EF even when accounting for use of antihypertensive medications (r = −0.132, *p* = 0.233). In group-wise analyses, however, CSP was the only parameter that correlated negatively with EF in the Control group, while no significant correlations were identified in the MFS group (Table 3).

Interestingly, even though none of the vascular parameters correlated with LV systolic or diastolic function in the MFS group, we did see a strong correlation of cfPWV with indexed LV mass (r = 0.604, *p* = 0.005) and LV volume (r = 0.581, *p* = 0.007). This was not seen in the control group (*p* > 0.2 for both). Linear regression analyses corrected for age and sex confirmed significant associations between cfPWV and LV volume index (B = 0.029 (95%CI 0.014–0.043), standardized beta 0.6, *p* < 0.001) as well as LV mass index (B = 0.028 (95%CI 0.009–0.048), standardized beta 0.5, *p* = 0.007) in the MFS group, but not in control group (*p* = 0.581 and *p* = 0.388, respectively; Figure 1B). In addition, cfPWV correlated strongly with BSP (r = 0.75, *p* < 0.001), BDP (r = 0.59, *p* = 0.007), and CSP (r = 0.5, *p* = 0.026) in the MFS group and only mild-moderately in the control group (r = 0.34–0.44, *p* < 0.005).

### Cardiovascular Function in Relation to Age

**FIGURE 2:**
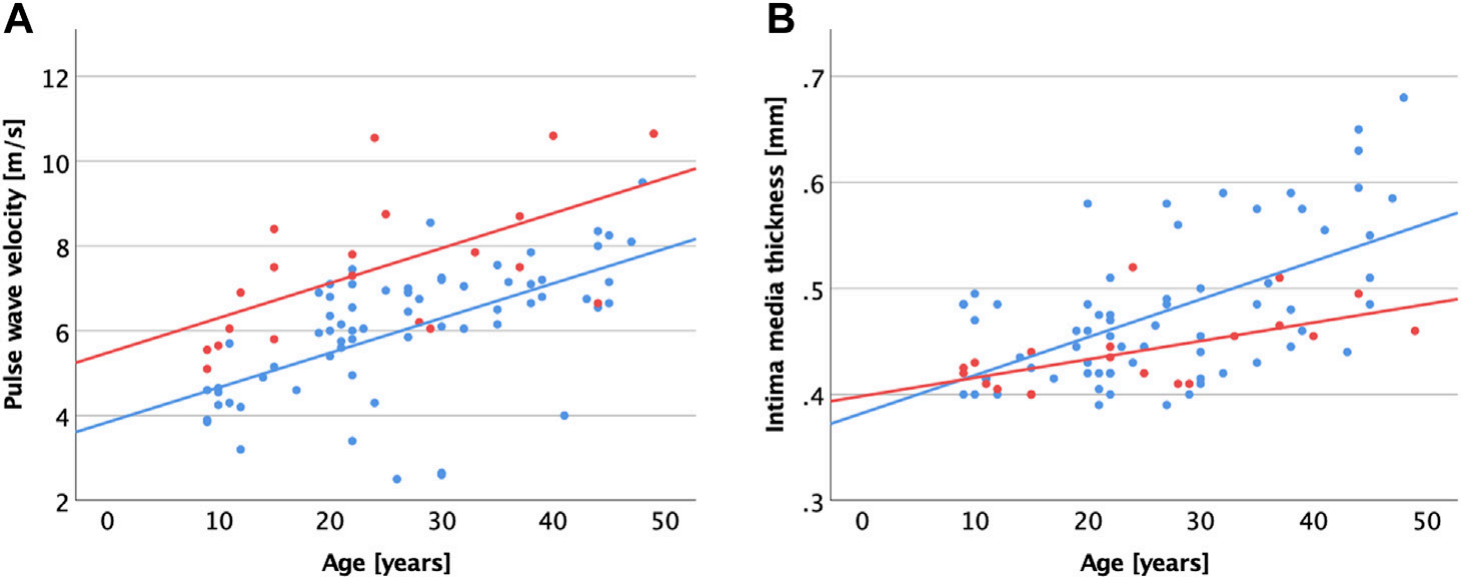
Scatter plot visualizing correlations between age and carotid-femoral pulse wave velocity **(A)**, common carotid intima media thickness **(B)** for patients with MFS (red dots and regression line) and healthy controls (blue dots and regression line).

Generally, chronological age correlated with vascular parameters and diastolic function, but not systolic function (Table 3). In the MFS cohort, cfPWV (Figure 2A) and cIMT (Figure 2B) correlated most strongly with age.

### Biomarkers

Sixteen MFS patients and 67 Controls had blood samples drawn which were analyzed for biomarkers. Linear regression analyses correcting for age and sex were performed. *p*-values were adjusted for multiple comparisons using the Benjamini–Hochberg correction. Brain Natriuretic Peptide (BNP) was the only biomarker significantly elevated in MFS patients (B = 0.581 (95%CI 0.271–0.890), *p* < 0.001; Table 4). Correlation analyses within the MFS group revealed that BNP correlates strongly and significantly with cfPWV (r = 0.680, *p* = 0.004), age (r = 0.648, *p* = 0.007), CSP (r = 0.565, *p* = 0.023), GLS (r = 0.551, *p* = 0.027), but not other characteristics of cardiac structure and function (e.g., EF: *p* = 0.691; LVEDVi: *p* = 0.565; LVMi: *p* = 0.125; E’: *p* = 0.583) or brachial blood pressure (BSP: *p* = 0.12; BDP: *p* = 0.194). Interestingly, none of the above mentioned associations were observed in the Control group (data not shown).

**TABLE 4:** Results from linear regressions models comparing each biomarker between MFS and controls, adjusting for age and sex.

Marker	β (95% CI)	p	Adjusted p^a^
bnp	0.581 (0.271; 0.890)	<0.001	0.040^b^
ren	0.462 (0.188; 0.736)	0.001	0.067
vegfd	0.252 (0.057; 0.447)	0.013	0.408
lpl	−0.231 (−0.447; −0.016)	0.039	0.677
boc	0.224 (0.002; 0.446)	0.052	0.677
itgb1bp2	−0.661 (−1.340; 0.017)	0.060	0.677
lep	0.593 (−0.029; 1.210)	0.066	0.677
cd40l	−0.551 (−1.150; 0.048)	0.075	0.677
prss27	0.234 (−0.027; 0.495)	0.083	0.677
il6	0.329 (−0.042; 0.701)	0.087	0.677
cxcl1	−0.527 (−1.130; 0.074)	0.090	0.677
lox1	0.277 (−0.047; 0.600)	0.098	0.677
src	−0.521 (−1.130; 0.090)	0.099	0.677
dkk1	−0.278 (−0.608; 0.052)	0.103	0.677
gal9	0.131 (−0.0400; 0.302)	0.137	0.771
ctrc	−0.298 (−0.701; 0.105)	0.151	0.771
hbegf	−0.193 (−0.457; 0.070)	0.155	0.771
tgm2	0.248 (−0.100; 0.596)	0.167	0.771
ho1	−0.145 (−0.352; 0.061)	0.172	0.771
mmp12	0.239 (−0.121; 0.599)	0.197	0.771

Only biomarkers with unadjusted *p* < 0.2 are presented.

a *p*-values adjusted with Benjamini & Hochberg (false discovery rate) correction.

b Marks all *p* < 0.05.

As renin achieved borderline significance with an adjusted *p* = 0.067, we added use of angiotensin II receptor blockers to the model, which confirmed that there is no difference between the groups (unadjusted *p* = 0.022).

## Discussion

In this prospective multimodal cardiovascular examination of 20 MFS patients, we demonstrate that MFS patients have generally increased aortic stiffness as evidenced by increased cfPWV in addition to AIx, AI, impaired ascending aortic elasticity, endothelial dysfunction, and BNP elevation. However, cIMT is decreased compared to controls, suggesting that the mechanism of general stiffening is different from acquired vascular disease.

### General Aortic Stiffening

As *FBN1*, the MFS disease gene, is ubiquitously expressed and the MFS phenotype manifests in several organs, it may not be surprising to see wide spread arterial changes that are not confined to the anatomically most affected location, i.e., the aortic root. Indeed, an increased PWV in MFS has been shown by multiple groups using multiple modalities, and accelerated arterial aging has been suggested particularly for the proximal aorta. (Westenberg et al., 2011; Salvi et al., 2018; Cui et al., 2021; Ruiz-Muñoz et al., 2021). This distinguishes MFS from Turner syndrome, another important genetic disorder. While Turner syndrome patients have increased focal aortic stiffness in the ascending aorta, cfPWV is not increased, arguing against general aortic stiffening in this systemic disease (Schäfer et al., 2018). Similarly in various types of congenital heart disease, increased arterial stiffness has been reported. (Müller et al., 2015; Häcker et al., 2018; Sandhu et al., 2021; Willinger et al., 2021). For patients with repaired CoA, there have been some reports on increased cfPWV. (Kenny et al., 2011; Madueme et al., 2013). However, cfPWV is blood pressure dependent, and when correcting for blood pressure, cfPWV does not appear to be increased in this group. (Weismann, Maretic et al., 2021). Similarly, others have demonstrated in patients with tetralogy of Fallot that PWV is increased in the ascending but not the descending aorta. (Saiki et al., 2012). When evaluating cfPWV and carotid-radial PWV though, there is no significant difference compared to controls. (de Groot et al., 2010). Thus, the extent of arterial stiffening in MFS is wider and distinct from that of structural CHD and Turner syndrome.

### IMT

To our knowledge, we have shown for the first time that cIMT may be decreased in MFS compared to controls while carotid artery elasticity appears to be similar to controls. Carotid artery remodeling in MFS so far has not been a focus of attention in the literature. However, decreased cIMT has been reported in patients with isolated mitral valve prolapse. (Erolu et al., 2018). The authors speculated that this may indicate a lower risk for atherosclerosis in these patients. In MFS, reduced carotid artery compliance has been demonstrated. (Kiotsekoglou et al., 2009). In addition, there is evidence that diminished aortic wall medial thickness may be linked to aortic dissection. (Shiran et al., 2014). Larger scale studies may investigate a possible role of carotid intima-medial thinning in predicting the risk for future aortic dissection.

### Endothelial Function

Endothelial dysfunction in MFS has been described in mouse models as well as in humans when evaluated using flow mediated vasodilation in the brachial artery (FMD). (Wilson et al., 1999; Chung et al., 2007; Syyong et al., 2009; Lomelí et al., 2018). In addition, a correlation of FMD with BSA ascending aortic but not aortic root dimension (normalized to BSA) has been suggested. (Takata et al., 2014). FMD is considered the gold standard for physiologic assessment of endothelial function, but the method is rather observer-dependent. We therefore chose EndoPAT—which measures endothelial function in the index finger with peripheral artery tonometry—as it produces observer-independent results and has been validated extensively in adults with coronary artery disease. (Matsuzawa et al., 2015) (Woo et al., 2014) To our knowledge, EndoPAT data in MFS has not been published previously. Following adjustment for age and sex, we demonstrate evidence of diminished endothelial function in this relatively small cohort of MFS patients. We did not see a correlation of RHI with aortic dimensions or Z-scores (data not shown). By contrast, other reports did not identify abnormalities in RHI (EndoPAT) in congenital heart disease patients. (de Divitiis et al., 2001; de Divitiis et al., 2003; Brili et al., 2005; Radke et al., 2014; Nozaki et al., 2018; Weismann, Ljungberg et al., 2021; Weismann, Maretic et al., 2021). This underlines that arterial physiology in MFS is distinct from CHD and acquired heart disease, where endothelial dysfunction and IMT thickening co-occur.

### Cardiac Function

While Marfan cardiomyopathy is a well described entity, the majority of the literature has focused on aortic manifestations. (Alpendurada et al., 2010). In wild-type mice, however, fibrillin-1 and collagen have been shown to be widely expressed in the heart. (Steijns et al., 2018). Moreover, MFS mice that are exposed to pressure overload develop an acute severe Erk1/2-Tgfb mediated cardiomyopathy that can be rescued by Losartan. (Rouf et al., 2017). In our small cohort of MFS patients, we see systolic and diastolic cardiac dysfunction as well as increased indexed LV volume and mass, which is in line with prior reports. (Alpendurada et al., 2010). In our cohort, increased LV mass can be explained by LV dilation. While we see no correlation of LV function with vascular parameters, LV volume and mass indices correlate strongly with cfPWV within the MFS group. In addition, BNP correlates strongly with both cfPWV (r = 0.68, *p* = 0.004) and CSP (r = 0.57, *p* = 0.023)—equivalent to pressure overload in the mouse model. (Rouf et al., 2017). As non-myocytes play a critical role in pathogenesis of MFS cardiomyopathy, we suggest that a decompensated Frank-Starling mechanism due to LV dilation may trigger then LV dysfunction. (Rouf et al., 2017). This may be why we see signs of structural remodeling and increasing BNP in correlation with increased arterial load first. Given that fibrillin-1 is widely expressed in the heart and aorta, MFS patients may be particularly sensitive to increased arterial load.

### Biomarkers

BNP and N-terminal pro-BNP (NT-proBNP) elevated in the setting of cardiac dysfunction as well as in response to increased preload or afterload. (Toischer et al., 2008). Clinically, they are commonly used biomarkers that e.g., help the emergency room clinician differentiate between cardiac and non-cardiac causes of dyspnoea. We have shown herein that BNP - measured as part of a panel with 92 biomarkers—is strongly associated with cfPWV, age, CSP, and GLS within the MFS group, but not in controls. Others have shown that NT-proBNP elevation above 214 pg/ml predicts risk for sudden arrhythmogenic cardiac death and sustained ventricular tachycardia in MFS. (Hoffmann et al., 2013). Interestingly, elevated NT-proBNP was also associated with older age, impaired left ventricular systolic and diastolic dysfunction in that study. While we showed strong correlations of BNP with age and GLS, we were unable to show a correlation with EF or diastolic function. This may well be due to the small sample size in our study. We speculate that increased aortic stiffness and afterload in MFS may lead to left ventricular dilation, which is supported by a strong correlation of cfPWV with BNP, LVEDV, and LVMi. Ultimately, this may lead to overt cardiac dysfunction and risk for life-threatening arrhythmias. Though proof of a causal relationship is beyond the scope of this study, we suggest that BNP, cfPWV, and CSP should be monitored routinely in MFS patients as they can be ameliorated pharmacologically. This approach may improve long-term outcome including risk reduction for life-threatening arrhythmias in MFS.

### Clinical Implications

Mouse data suggests that fibrillin-1 is widely expressed in the heart and aorta, and that MFS mice are particularly sensitive to increased arterial load, which leads to a pressure induced cardiomyopathy that can be prevented by Losartan. (Rouf et al., 2017; Steijns et al., 2018). Our data show that cfPWV, a marker of general aortic stiffening, correlates with markers of cardiac stress (BNP and LV size) as well as blood pressure in MFS patients. We suggest that clinical management should include strict blood pressure control using ARBs, and that cfPWV, BNP, and LV size be monitored in MFS patients.

### Limitations

An important limitation of this study is that the MFS cohort is rather small, as it is a rare disease, and this is a single center study. We were unable to demonstrate a correlation of arterial parameters with LV function. This may be due to a combination of small sample size, diverse clinical backgrounds as well as pharmacologic treatment regimens. As 25% of MFS patients in our cohort had previously undergone aortic root replacement, it was not possible to perform correlation analyses between aortic dimensions and outcome variables as the groups size would have been too small. The cross-sectional design of the current study does not allow prognostication of adverse outcome.

In conclusion, MFS patients appear to have generally increased aortic stiffness and endothelial dysfunction, but decreased cIMT compared to controls, supporting that the mechanism of arterial stiffening is different from acquired vascular disease and other congenital cardiac lesions such as aortic coarctation. CfPWV is associated cardiac size, blood pressure, and BNP in MFS patients. These may be early markers of disease progression that are suitable for monitoring pharmacological treatment effects in MFS patients.

## Data Availability

The raw data supporting the conclusion of this article will be made available by the authors, without undue reservation.
